# P04 Low-dose polymyxin B is non-inferior to conventional doses in dialysis dependent and non-dialysis patients with Gram-negative sepsis: a real-world propensity-score matched cohort study

**DOI:** 10.1093/jacamr/dlaf230.011

**Published:** 2025-12-04

**Authors:** Asha K Rajan, Vishal Shanbhag, Vijayanarayana Kunhikatta, Ravindra Prabhu Attur, Girish Thunga

**Affiliations:** Department of Pharmacy Practice, Manipal College of Pharmaceutical Sciences, Manipal Academy of Higher Education, Manipal, Karnataka, India; Department of Critical Care Medicine, Kasturba Medical College, Manipal Academy of Higher Education, Manipal, Karnataka, India; Department of Pharmacy Practice, Manipal College of Pharmaceutical Sciences, Manipal Academy of Higher Education, Manipal, Karnataka, India; Department of Nephrology, Kasturba Medical College, Manipal Academy of Higher Education, Manipal, Karnataka, India; Department of Pharmacy Practice, Manipal College of Pharmaceutical Sciences, Manipal Academy of Higher Education, Manipal, Karnataka, India

## Abstract

**Background:**

Polymyxin B remains a key treatment option for infections caused by MDR Gram-negative bacilli, particularly in critically ill patients. However, its optimal dosing strategy recommendation remains uncertain, especially in those undergoing renal replacement therapy.

**Objectives:**

To compare the clinical and microbiological outcomes of low, usual and high-dose polymyxin B in a real-world ICU population.

**Methods:**

This 5 year retrospective cohort study included critically ill adult patients with Gram-negative sepsis who received polymyxin B. Patients were categorized into low-, usual- and high-dose groups based on loading and total daily maintenance dose. Pairwise propensity score matching was performed to adjust for baseline differences. The primary outcome was 28 day all-cause mortality. Secondary outcomes included microbiological clearance, ventilator-free days, ICU-free days and vasopressor-free days. Subgroup and sensitivity analyses were conducted, including within patients requiring dialysis. All the statistical analysis was performed using R software.

**Results:**

A total of 674 patients were included. After matching, usual-dose polymyxin B was associated with significantly higher 28 day mortality compared to the low-dose group (HR: 1.47 [1.11–1.95]; *P*=0.007). Vasopressor, ventilator and ICU-free days were also significantly higher in the low-dose group. No significant survival advantage was observed with high-dose regimens. Among dialysis-dependent patients (*n*=254), mortality did not differ significantly across dosing groups, though microbiological clearance was better with low dosing. Sensitivity and subgroup analysis also supported the robustness of the results.

**Conclusions:**

Low-dose polymyxin B was found to be non-inferior to the conventional dose in terms of clinical outcomes, without compromising microbiological efficacy. Supplemental dose offered no added benefit in patients undergoing dialysis. These findings support the use of individualized, lower dosing strategies, particularly in patients with renal impairment. Prospective trials are needed to define the optimal dosing threshold balancing efficacy and safety.

